# Paraoxonase-1 and Simvastatin Treatment in Patients with Stable Coronary Artery Disease

**DOI:** 10.1155/2016/6312478

**Published:** 2016-04-11

**Authors:** Rafał Januszek

**Affiliations:** Department of Cardiology, University Hospital, Kopernika Street 17, 31-501 Cracow, Poland

## Abstract

*Background.* Paraoxonase-1 (PON1) is the crucial antioxidant marker of high-density lipoproteins. The present study is aimed at assessing the effect of simvastatin treatment on PON1 activity and its relationship to Q192R and M55L polymorphisms in subjects with stable coronary artery disease (CAD).* Methods.* The patient group was composed of 53 individuals with stable CAD, and the control group included 53 sex-matched police officers without CAD. CAD patients were treated with simvastatin 40mg/day for 12 months. Respectively, flow mediated dilatation (FMD), serum hs-CRP and TNF-*α* levels, urinary 8-iso-PGF_2*α*_ concentrations, and PON1 activity were evaluated in definitive intervals.* Results.* There was no effect of simvastatin treatment on urinary 8-iso-PGF_2*α*_. Simvastatin treatment significantly increased FMD value, decreased CRP and TNF-*α* concentration. After adjusting for PON1 genotypes, significantly higher PON1 activity was noted in the 192R allele carriers, in both groups. Regardless of genotype, PON1 activity remained stable after simvastatin treatment.* Conclusions.* The present study confirms a positive effect of simvastatin therapy on endothelial function and inflammatory markers in secondary prevention. Simvastatin treatment shows no effects on PON1 activity and 8-isoprostanes level. The effect of simvastatin therapy on PON1 activity is not modulated by Q192R and M55L polymorphisms.

## 1. Introduction

Statins reversibly inhibit HMG-CoA reductase, also attenuating production of important isoprenoid intermediates used for posttranslational modification of different proteins [1]. The main pleiotropic effects of statin therapy are improvement of endothelial function, stabilisation of atherosclerotic plaques, anti-inflammatory properties, antithrombotic activity, and antioxidative effects [2]. All paraoxonases are lactonases, characterised by the widest known substrate specificity, and, due to the involvement in various signaling pathways, they play an important role in the anti-inflammatory and antioxidant response [3]. Paraoxonase-1 (PON1) is the first described esterase that hydrolyzes pesticides as toxic metabolites of parathion-paraoxon [4]. PON1 is almost exclusively present in HDL particles [5]. Peroxidase and esterase PON1 activity are crucial in detoxifying oxidative stress mediators, whereas lactonase activity is responsible for the hydrolysis of various compounds. Furthermore, PON1 presenting esterase activity hydrolyzes the ester bond present in oxidized cholesterol esters, thereby scavenging oxidized fats from atherosclerotic lesions [6]. In PON1 knockout mice, it has been shown that HDL fails to prevent oxidation of LDL and monocyte chemotaxis. On the other hand, the overexpression of PON1 in transgenic mice increased the effect of HDL on oxidation and attenuated the rate of formation of atherosclerotic lesions [7, 8]. Five polymorphisms were recognized in the PON1 gene promoter region. Some of the polymorphisms significantly affect gene expression and impact serum PON1 concentration. Currently there are two known polymorphisms within the coding region of PON1, which leads to the conversion of glutamine→arginine at position 192 (Q192R) and leucine→methionine at position 55 (L55M). The genetic polymorphisms of PON1 gene influence PON1 enzyme concentration and specific activity. The Q192R polymorphism does not affect the serum PON1 concentration but significantly modifies its activity. 192R carriers show the highest activity of PON1 against paraxone, whereas 192QQ homozygotes show the highest PON1 activity against phenylacetate [9]. The L55M polymorphism mostly influences binding affinity of HDL and PON1 [10]. Homozygotes 192QQ and 55MM show the highest protection from LDL oxidation, while 192RR and 55LL homozygotes show LDL with the least resistance to oxidation [11]. Genetic factors, including polymorphisms, are responsible for more than 60% of the phenotypic variability of PON1. Demographic factors such as age and sex account for 1–6% of PON1 variability, while metabolic variables account for about 4–19% [12]. Eating habits, physical activity, and smoking also modify serum PON1 activity and oxidative stress [13]. Thus far, published studies highlighted that statins can have a beneficial influence not only by their lipid-lowering effect, but also through diminishing oxidative stress and enhancing PON1 activity. The improvement in PON1 activity after statin therapy could be generated by oxidative stress reduction, a direct effect on promoter activity and PON1 gene expression [14]. Additionally, patients with coronary artery disease (CAD) present lower serum PON1 activity. This was found to be a predictor of CAD, although not all studies have confirmed this relationship [15]. A weak association of CAD with Q192R polymorphism was demonstrated in prior research [16]. The leading inflammation marker is high sensitivity C-reactive protein (hs-CRP), which is substantially regulated by tumor necrosis factor *α* (TNF-*α*). The increased expression of TNF-*α* intensifies production of reactive oxygen species (ROS) and decreases bioavailability of NO, leading to endothelial dysfunction [17]. Statins present anti-inflammatory properties. However, the effect of statins on fibrinogen is inconsistent [18, 19]. Risk factors of atherosclerosis enhance expression of NADPH oxidase, leading to significant generation of O_2_^−^ and, consequently, depletion of NO bioavailability. Statins improve endothelial function in patients with hypercholesterolemia and atherosclerosis [20]. The “free radical chain reaction” initiated by ROS results in the formation of additional reactive compounds, including F_2*α*_-isoprostanes which is largely represented by 8-iso-prostaglandin-F_2*α*_ (8-iso-PGF_2*α*_). Higher concentrations of 8-iso-PGF_2*α*_ and its metabolites were demonstrated in patients with CAD, and 8-iso-PGF_2*α*_ is a marker of CAD [21]. Statins decrease oxidative stress by inhibition of oxidative stress in the endothelium, their effects on cardiac muscle cells, and their influence on PON1 activity. At this time, the dominating theory is that statin therapy reduces oxidative stress markers [22]. The mechanism of PON1's protective effects seems to be tightly associated with the antioxidant potential of the enzyme. PON1 scavenges oxidative stress products, leading to improved endothelial function, especially in patients with CAD. The effect of statin therapy on PON1 activity in CAD patients treated with simvastatin for 12 months has yet to be evaluated, while shorter studies did not provide conclusive results. Also, research assessing the relationship of Q192R and L55M polymorphisms with CAD produced inconclusive results.

The main goal of the present study was to assess the effect of 12 months of simvastatin treatment on serum PON1 activity and urinary 8-isoprostanes in patients with CAD. The present study also aimed to evaluate the association of Q192R and L55M PON1 gene polymorphisms on PON1 activity with and without simvastatin treatment. The effect of simvastatin treatment on endothelial function, intima-media thickness (IMT), proinflammatory marker concentrations, and lipid profile was also assessed to confirm parallel simvastatin efficacy.

## 2. Subjects and Methods

### 2.1. Subjects

The present study was performed on 53 patients, aged 35 to 65 years with stable CAD (based on imaging study, angiocomputed tomography of coronary arteries or coronarography, echocardiography, and exercise treadmill test with electrocardiogram). The control group consisted of 53 active, professional police officers without CAD. Twenty-two obese individuals were included in the patients group and 18 in the control group. All participants of the present study qualified for the simvastatin therapy were permitted to continue treatment with aspirin 75–100 mg/day, *β*-blockers, angiotensin-converting enzyme inhibitors, calcium channel blockers, diuretics, and nitrates, at a stable dose throughout the study period. Some participants in the control group remained on treatment due to concomitant diseases. Patients who required a change in chronic therapy during the study period were excluded from the study. Patients were qualified for simvastatin treatment according to the current ESC guidelines. Individuals who had an acute coronary episode in the last 3 months, heart failure, LDL < 1.8 mmol/L, and triglycerides level > 4.5 mmol/L and were previously treated with lipid-lowering agents or oral anticoagulants were excluded from the study. Also, patients with recently peracted infections, diabetes, impaired renal and liver function, myopathy, and neoplastic and thyroid disease were not included in the study. Participants with CAD were treated with simvastatin 40 mg daily for 12 months. Throughout the present study, participants were restricted from nonsteroidal anti-inflammatory drugs and maintained a stable level of physical activity and diet. The percentage of smokers in the two groups also did not change significantly during the present study. The lipid profile, flow-mediated dilation (FMD), intima-media thickness (IMT), serum fibrinogen, CRP, and TNF-*α*, as well as urine 8-iso-PGF_2*α*_ and serum PON1 activity, were evaluated at baseline, after 6 and 12 months of simvastatin treatment in the CAD group, and at baseline and after 12 months in the control group. PON1 polymorphisms (Q192R and M55L) were assessed at baseline in all participants. Blood samples were collected after a 14-hour overnight fast, and FMD measurements were assessed the following day. Patients were asked to refrain from smoking 12 hours prior to the blood test. Blood samples for the analysis of fibrinogen, hs-CRP, lipid profile, and creatinine were drawn from the forearm vein (between 6.00 and 8.00 a.m.) after 30 min rest and immediately sent to the laboratory. Blood and urine samples for further analysis (PON1 activity, PON1 gene polymorphisms, TNF-*α*, urine 8-iso-PGF_2*α*_, and creatinine) were frozen and stored at minus 80 degrees centigrade. No signs of myopathy or liver function impairment were observed during the study. The present study was approved by the local Ethics Committee and conducted according to the ethical standards outlined in the Declaration of Helsinki and its amendments. All participants of the present study signed a written informed letter of consent.

### 2.2. Imaging Studies

The FMD measurement was performed according to the previously published protocol [23]. The mean IMT value was established on the basis of three ultrasound measurements in the distal section of the common carotid artery, approximately 2 cm from the bifurcation, at cross-sectional and longitudinal sections, and in the anterolateral and posterolateral presentations.

### 2.3. PON1 Activity and Polymorphisms

Serum PON1 activity was determined spectrophotometrically using a spectrophotometer 8730UV PU/VIS. Paraoxon hydrolysis rate to p-nitrophenol was monitored (*ε* = 13 000^−1^ cm^−1^) in K-HEPES buffer, at 28°C at length 412 nm. One unit of PON1 catalyzes the formation of 33.5 pmol of p-nitrophenol in 10 minutes at 25°C with 0.5 *μ*L of serum in 0.5 mL of the reaction mixture (equivalent to 0.001 A_412_). The L55M and Q192R polymorphisms in the PON1 coding gene were tested by analysis of restricted fragment length polymorphism (RFLP) of amplified gene segment by polymerase chain reaction (PCR). PON1 genetic variants that cause amino acid substitution Q192R and M55L were genotyped by PCR amplification and further restriction digestion (PCR-RFLP) with section digestion on an agarose gel. Replacement of glutamine (Q) to arginine (R) at position 192 of the polypeptide chain is due to the transition of adenine to guanine, detected by restriction digestion using the MboI enzyme. Genomic DNA fragment of 291 bp in length was amplified under standard conditions of 34 cycles using primers with the following sequence: 5′-ATA GAA GAG GAC AGT CAG TGC TT and 5′-CAT CAT CGG TGC ACT GTG AA TT. In the case of the AA genotype (homozygous 192QQ), the size of digestion products was 154 and 137 base pairs. For the GG genotype (homozygous 192RR), the length of the digestion products was 137, 126, and 28 base pairs. Heterozygote (AG 192QR) had a combination of these passages, 154, 137, 126, and 28 base pairs. Replacement of methionine (M) to leucine (L) at position 55 in the amino acid chain results from the substitution of adenine to thymine, detected with the restriction enzyme NlaIII. Genomic DNA with a length of 293 bp was amplified under standard conditions using primers of appropriate sequences: 5′-TTT CCA CGC TAT AAT CAT ATT C and 5′-AAT TTA AAC GAC TGC TCC CAG TA. In the case of the AA genotype (homozygous 55MM), the size of digestion products was 192 and 101 base pairs. In the case of TT genotype (homozygous 55LL), amplified fragment was not subject to restrictase cutting, and its length was 293 bp. Heterozygotes 55ML had a combination of all of these fragments, although largely 293, 192, and 101 base pairs.

### 2.4. Markers of Inflammation and Oxidative Stress

Serum ultrasensitive CRP protein was determined by nephelometry (*Dade-Behring, Germany*). TNF-*α* levels were measured by ELISA (*R&D Systems, United Kingdom*). Plasma fibrinogen concentration was determined by the use of BCT coagulometer (*Behring Coagulation Timer, Dade-Behring, Germany*) and Multifibren U analyzer. Urine 8-iso-PGF_2*α*_ concentrations were determined by ELISA (*EIA Kit Urinary Isoprostane, NucliLab BV, Netherlands*).

### 2.5. Lipids

Total cholesterol and triglycerides were determined by an enzymatic method (Technicon RA-1000 analyzer).

### 2.6. Statistical Analysis

STATISTICA for Windows Release 10 (Statsoft Inc., 2011) was used for data analysis. The test choice depended on the distribution of particular data. To compare measurable variables (or to assess the statistical significance of the observed differences), parametric tests were used: the two-sided Student's *t*-test and Spearman linear correlation. In case of missing mentioned assumptions, nonparametric tests were used (Friedman ANOVA, Mann-Whitney *U* test, Kruskal-Wallis, and Chi^2^ test). Multivariate analysis of variance was also performed. *p* values < 0.05 were considered significant.

## 3. Results

### 3.1. Baseline Characteristics of the Study Groups

Table 1 shows clinical characteristics of the investigated groups.

### 3.2. Effects of Simvastatin Treatment on Lipid Profile

At baseline, significantly higher total (*p* = 0.00004) and LDL cholesterol levels (*p* < 0.000001) were found in the patient group. No significant differences were demonstrated between patient and control groups in HDL cholesterol and triglycerides concentrations. The effect of simvastatin treatment on lipid profile is presented in Table 2.

### 3.3. Proinflammatory Markers

At baseline, serum hs-CRP and TNF-*α* concentration in the CAD group were significantly higher compared to the control group (*p* = 0.0005 and *p* = 0.00002). In the control group, the mean fibrinogen concentration was similar in both time points and was significantly lower than in the patient group (*p* = 0.02, *p* = 0.005). The effect of simvastatin treatment on hs-CRP, TNF-*α*, and fibrinogen concentration is presented in Table 2.

### 3.4. The Urine 8-Iso-PGF_2*α*_

Initially, the urine 8-iso-PGF_2*α*_ concentration was significantly higher in the treated group (*p* < 0.0001). Furthermore, after 12 months of simvastatin treatment, the urine 8-iso-PGF_2*α*_ concentration in the patient group remained significantly higher compared to that in the control group (*p* < 0.0001). The effect of simvastatin treatment on urine 8-iso-PGF_2*α*_ is presented in Table 2.

### 3.5. Endothelial Function and Intima-Media Thickness

At baseline, the mean FMD was significantly lower in the patient group in comparison to the control group (*p* = 0.015). Compared to the baseline, there was a significant increase of the FMD after 6 (*p* = 0.0001) and 12 months (*p* = 0.006) of treatment. The FMD values observed after 6 and 12 months of treatment with simvastatin in the patient group were similar to those found in the control group. In the patient group, the FMD negatively correlated with age (*p* = 0.03) and IMT (*p* = 0.002) and correlated weakly with hs-CRP (*p* = 0.02). At baseline, IMT values were significantly higher in the patient group (*p* < 0.000001). In the patient group, significant reduction of IMT after 6 and 12 months of simvastatin treatment was observed (*p* = 0.008 and *p* = 0.004), although IMT still remained higher after treatment compared to controls (*p* = 0.003). It was also found that IMT correlated positively with age (*p* = 0.0002) and negatively with FMD (*p* = 0.002; Table 2). Multivariate ANOVA analysis confirmed the significance of simvastatin treatment to decrease lipid profiles (total and LDL cholesterol), hs-CRP concentration, and IMT. ANOVA also showed a slight reduction in TNF-*α* concentration, but no reduction in FMD.

### 3.6. Genetic Polymorphisms and PON1 Activity

**Figure 1:**
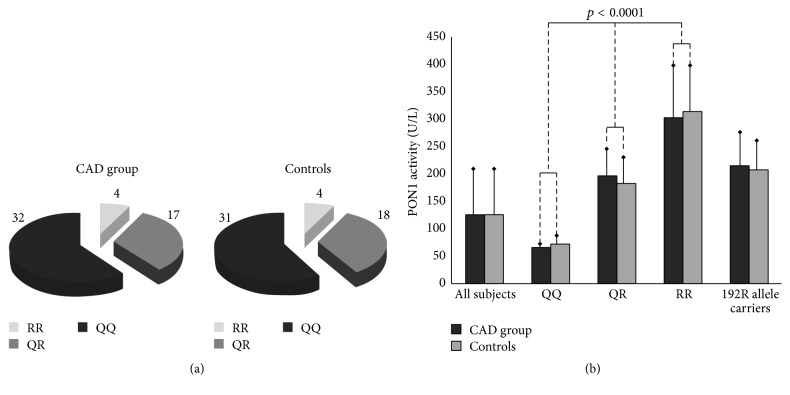
The L55M polymorphism of the PON1 gene: (a) the percentage distribution of PON1 genotypes in patient and control groups; (b) the association of Q192R polymorphism with serum PON1 activity.

**Figure 2:**
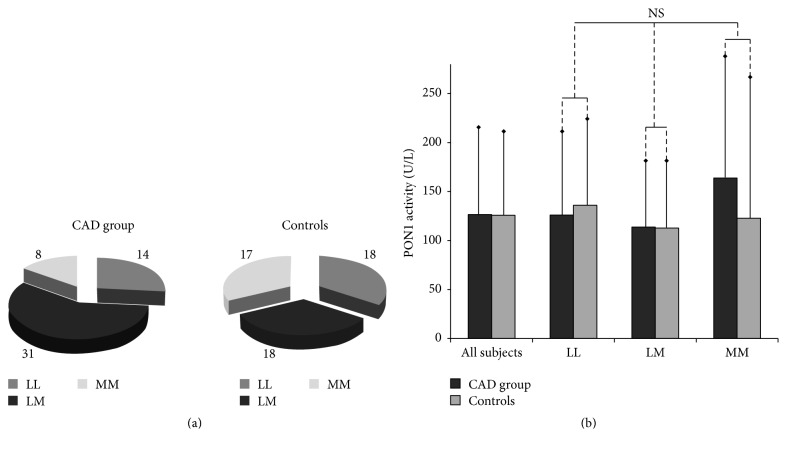
The Q192R polymorphism of the PON1 gene: (a) the percentage distribution of PON1 genotypes in the patient and control groups; (b) the association of Q192R polymorphism with serum PON1 activity.

No deviation from genetic equilibrium in Q192R polymorphism was observed (according to the Hardy-Weinberg principle), both in the patient and in control groups (*p* = 0.81; *p* = 0.13). Similar percentage distribution of the particular genotypes was noticed in the patient and control groups (Figure 1, Table 3). Also, frequency of the 192R allele was similar in both groups (0.235 in patients and 0.245 in controls), although genetic deviation from equilibrium of L55M polymorphism was observed in the control group (*p* = 0.01). The 55LL genotype was found in 26.4% and 34% of individuals, 55LM in 58.5% and 34%, and 55MM in 15.1% and 32% (in patients and controls, resp.; Figure 2, Table 3). At baseline, the average serum PON1 activity in the patient group was comparable to that in the control group (Table 4). No significant differences were found between the patient and control groups regarding PON1 activity in different genotypes (*p* = 0.14; *p* = 0.43; *p* = 0.85 for genotypes 192QQ, 192QR, and 192RR, resp., Table 3). Higher PON1 activity was found in patients with 192RR genotype as compared to heterozygotes 192QR and homozygotes 192QQ (*p* < 0.0001, Figure 1, Table 3). Due to the small number of individuals with genotype 192RR, they were included in the group with genotype 192QR, creating a “192R allele carriers” group. The average PON1 activity in 192R allele carriers was significantly higher compared to those with genotype 192QQ (*p* < 0.0001 and *p* < 0.0001, in patients and controls, resp., Table 3, Figure 1). Table 4 presents the effect of simvastatin treatment on serum PON1 activity according to genotype. After 6 and 12 months of treatment with simvastatin, the mean PON1 activity did not change significantly compared to baseline values in each genotype. In the control group, the average PON1 activity was similar at baseline and after 12 months. There were no significant differences in PON1 activity between individuals with genotype 55LL, 55LM, or 55MM in both patient and control groups (Table 3). PON1 activity in each genotype group for the L55M polymorphism did not differ significantly between individuals in patient and control groups (Figure 2, Table 3). Patients in each genotypic group (55LL, 55LM, and 55MM) showed no significant changes in PON1 activity after 6 and 12 months of simvastatin treatment when compared to baseline (Table 4). In controls, the average PON1 activity was similar at baseline and after 12 months in each genotypic group (Table 4). PON1 activity after simvastatin treatment was analyzed to examine the impact of lifestyle choices and genetic polymorphisms among participants. Analysis revealed no significant differences in PON1 activity after simvastatin treatment among smokers and nonsmokers and obese and nonobese participants and after adjusting for both L55M and Q192R polymorphisms.

Analysis of all study participants revealed all 9 possible PON1 gene combination genotypes (Table 5). Considering both of the analysed PON1 gene polymorphisms, significantly higher (*p* < 0.0001) initial PON1 activity was found in patients with combined genotype containing 192R allele (192QR/55LL, 192QR/55LM, 192QR/55MM, 192RR/55LL, 192RR/55LM, and 192RR/55MM), compared to Q allele homozygotes (192QQ/55LL, 192QQ/55LM, and 192QQ/55MM). There were no significant differences between PON1 activities at baseline and after 6 and 12 months of treatment with simvastatin, in patients with combined genotypes containing 192R allele. The lowest PON1 activity, in both patients and control group, was found in individuals with the combined genotype 192QQ/55MM, compared to genotypes 192QQ/55LL and 192QQ/55LM. After 6 months of treatment with simvastatin, significant decreases in PON1 activity were only observed in individuals from the patient group with combined genotype 192QQ/55LM (*p* = 0.007) (Table 5).

## 4. Discussion

### 4.1. Simvastatin Treatment and Lipid Profile

In the present study, simvastatin significantly reduced total cholesterol (by 24.5% and 17.4%) and LDL (by 37.7% and 24.8%) levels after 6 and 12 months of treatment. Similar results were published in large randomized trials, although a greater lipid-lowering effect was observed in trials compared with the present study [24]. A relatively low initial mean cardiovascular risk and low baseline total cholesterol and LDL undoubtedly influenced the lipid-lowering effect of simvastatin in the present study.

### 4.2. Extra-Lipid Effects of Simvastatin Therapy

Simvastatin treatment significantly reduced hs-CRP and TNF-*α* levels which confirmed pleiotropic properties of statins. The effect of statin therapy on fibrinogen is still not established [25]. Currently, there are no specific cut-off points for urine F_2*α*_-isoprostane levels. Analysing the results of published studies, we observed a large variability in the range of F_2*α*_-isoprostane concentrations, which may be affected by measurement methods, the specific substrate used, the substance investigated, and coexisting medications and diseases. The 8-iso-PGF_2*α*_ values in the present study were higher compared to one publication and comparable to other papers [26, 27]. Most of the published studies found that statin therapy reduced concentration of 8-iso-PGF_2*α*_. However, many of these studies were conducted on small populations with hypercholesterolemia and excluded atherosclerosis [28]. The present results remain in line with results reported by other researchers [29]. Perhaps, simvastatin therapy has not been efficient enough to prevent lipid peroxidation and indirectly the burden of atherosclerosis. It has been previously proven that statins improve FMD in patients with stable angina [30]. A recently published large meta-analysis showed that statins have a significant effect on IMT in secondary prevention, but not in primary prevention, which we found as well in the present study [31].

### 4.3. PON1 Activity and CAD

The most convincing evidence for the PON1's association with atherosclerosis was discovered with transgenic mice [8]. Human studies also indicated that low serum PON1 activity is a predictor of adverse cardiovascular events. However, conflicting results were published shortly thereafter [32, 33]. Currently, the prevailing view is that PON1 activity is lower in patients with CAD [32]. The Q192R polymorphism does not influence serum concentrations of PON1 but affects its activity for a specific substrate. The highest activity against paraoxon occurs in 192R allele carriers, while the highest activity against phenylacetate occurs in individuals with genotype 192QQ [12]. In the present study, paraoxon was used as a substrate for PON1 activity assessment, and the highest activity was found in the group of 192R allele carriers. Analysis of combined genotypes confirmed the effect of 192R allele influence on PON1 serum activity. The average PON1 activity in CAD patients and in different genotype groups was comparable to that in controls, which is consistent with recently published research [33]. In most case-control studies, participants in the control group were younger than patients in treatment groups. However, few studies investigated the effect of age on serum PON1 activity [34]. The relatively small size of the study groups affected the results. Our results show no association of the L55M polymorphism with serum PON1 activity and CAD risk. This conclusion is supported by the results of a large meta-analysis which found only a weak association of Q192R polymorphism with CAD [35].

### 4.4. The Genetic Polymorphisms of PON1 and Simvastatin Effects

At present, little is known about the pharmacological regulation of PON1 in humans. Antioxidant properties of statins demonstrated on animals were also verified in human studies, where individuals with hypercholesterolemia presented significant reduction in oxidative stress markers and increased PON1 activity after statin therapy [36]. The increase in PON1 activity is the consequence of oxidative stress reduction, enhanced expression, and attenuated inactivation of PON1. These results were based on research demonstrating a direct effect of statin therapy on the promoter activity and expression of the PON1 gene [37]. It has been shown that sterol regulatory element-binding proteins (SREBP-2) increase promoter region activity proportionally to the statin dose, suggesting a relationship between PON1, intracellular cholesterol homeostasis, and lipid metabolism. Statins additionally inhibit isoprenoid mediators which influence PON1 activity. Statins increase the activity of an enzyme associated with HDL and PON1/HDL ratio [38]. However, in the present study there was no effect of simvastatin on PON1 activity in the patient group, neither before nor after adjusting PON1 activity for the Q192R polymorphism. Maintained elevation of the urine 8-iso-PGF_2*α*_ may explain why our results show that PON1 activity did not increase after simvastatin treatment. Regarding published data, it should be noted that it is difficult to compare particular results due to differences in the assay methods, investigated populations, selection bias, and ethnic differences. Inclusion criteria of participants often did not address smoking, obesity, or age, and the number of the participants was usually small. Only a few studies focused on the effect of statins on PON1 activity in patients with CAD, and they revealed increases in HDL concentration and PON1 activity [39]. Aspirin treatment could influence our results. In contrast to published works, all individuals from the patient group were treated with aspirin at the dose of 75 mg/day. Aspirin's dose-dependent inhibitory effect on the formation of p-nitrophenol indicates that aspirin is also hydrolyzed with paraoxon and may compete for the active site of PON1 [40]. Furthermore, aspirin hydrolysis to salicylic acid occurred under the influence of isolated HDL showing that, in addition to hepatocytic hydrolysis, aspirin is also hydrolyzed in circulation. Based on this data, it could be concluded that results are underestimated in patients treated with aspirin, especially when paraoxon is used for the assessment of PON1 activity. Although this hypothesis requires further studies, deactivation changes of the enzyme induced by acetylation of tyrosine in the active center of PON1 could not be excluded. In another study, which focused on aspirin's relation to PON1 activity in patients with CAD, higher PON1 activity was demonstrated in individuals treated with aspirin, although the results and PON1 activity were not adjusted to evaluate the Q192R polymorphism [41]. On the other hand, Waterman et al. found prooxidative properties of aspirin at dose of 75 mg/day in healthy volunteers increased* ex vivo* sensitivity of LDL to oxidative modification, triggering the growth of proatherogenic properties of LDL cholesterol. The oxidative stress hypothesis is supported by evidence linking oxidative stress with aspirin and PON1 [42]. An alternative thesis is an anti-inflammatory activity of aspirin, though the PON1 concentration is reduced during an inflammatory response in animal models [43]. Daily aspirin therapy was introduced in most participants 2-3 weeks before initiating simvastatin therapy. Perhaps increased PON1 activity after aspirin therapy masked the expected difference in PON1 activity between patients and controls. The potential improvement of PON1 enzyme activity under the influence of pharmacological agents is presumably low, around 7–11% [36]. Hypothetically assuming that PON1 activity was to improve after aspirin treatment, further improvement after simvastatin may have been imperceptible. We cannot exclude that the effect of statins was attenuated by the prooxidative effect of aspirin or other factors associated with CAD, which decreased PON1 activity. Plasma PON1 concentration was not measured in the present study. This is a methodological imperfection and may have significance in determining the effect of statins on PON1 activity. Additionally, the atheroprotective effects of PON1 activity may vary considerably if PON1 reacts with a compound other than paraoxon substrates, such as lipid hydroperoxides. Hydrolysis of paraoxon* in vitro*, which is an expression of PON1 activity, may be a suboptimal method of measuring its current antioxidant capacity. It is possible that the use of different assays to determine PON1 activity could give dissimilar results. Thus far, there is no known satisfactory method for accurate measurement of the antioxidant potential of PON1* in vivo*. The quantification of isoprostanes by gas chromatography/mass spectrometry could also amend the results.

In conclusion, the present study supports the theory that the effect of 12 months of simvastatin treatment on PON1 activity and urine 8-isoprostanes is not significant in patients with stable coronary artery disease. The positive effect of simvastatin treatment on PON1 activity and reduction in oxidative stress markers could be disguised by other factors which reduce the effectiveness of simvastatin, such as coexisting medical treatment, atherosclerosis burden, or coexisting risk factors for atherosclerosis.

The decrease of inflammatory markers, intima-media thickness, and improvement of endothelial function after simvastatin treatment confirms the pleiotropic effects of simvastatin in patients with stable CAD.

Q192R and M55L PON1 gene polymorphisms are not associated with an altered effect of simvastatin treatment on PON1 activity and urine 8-isoprostanes in patients with stable CAD treated for 12 months with simvastatin. The R allele of Q192R polymorphism is associated with higher PON1 activity in patients with CAD. Neither Q192R nor M55L PON1 gene polymorphism was associated with increased coronary artery disease incidence.

## Figures and Tables

**Table 1:** Characteristics of the study groups.

	CAD group	Controls	*p*
Number of patients; females	53 (9)	53 (6)	0.4^*∗∗*^
Age; years	51.3 ± 7.8 (38 ÷ 65)	42.03 ± 4.82 (35 ÷ 55)	<0.000001^*∗*^
Framingham Risk Score; %	12.1 ± 6.4 (1.8 ÷ 28)	4.9 ± 3.6 (0.2 ÷ 19.8)	<0.000001^*∗*^
BMI; kg/m^2^	29.4 ± 4.9 (19.5 ÷ 44.7)	29.2 ± 3.8 (21.7 ÷ 40.7)	0.81^*∗*^
Waist-hip ratio	0.96 ± 0.06 (0.77 ÷ 1.09)	0.94 ± 0.06 (0.74 ÷ 1.1)	0.19^*∗*^
Overweight; *n* (%)	20 (37.73)	28 (52.83)	0.11^*∗∗*^
Obesity; *n* (%)	22 (41.45)	18 (33.96)	0.72^*∗∗*^
Hypertension; *n* (%)	37 (69.81)	19 (35.84)	0.0005^*∗∗*^
CAD; *n* (%)	53 (100)	—	—
History of			
Myocardial infarction; *n* (%)	7 (13.2)	—	—
PCI; *n* (%)	9 (16.98)	—	—
CABG; *n* (%)	1 (1.88)	—	—
Smoking history			
Whenever; *n* (%)	35 (66.04)	27 (50.94)	0.52^*∗∗*^
Number of pack-years	25.75 ± 9.04 (1 ÷ 40)	15.25 ± 7.04 (1 ÷ 26)	0.007^*∗*^
Currently; *n* (%)	17 (32.07)	16 (30.18)	0.83^*∗∗*^
COPD; *n* (%)	6 (11.32)	0	0.01^*∗∗*^
Familial history of cardiovascular diseases; *n* (%)	40 (75.47)	29 (54.71)	0.02^*∗∗*^
Medications			
Aspirin; *n* (%)	53 (100)	7 (13.2)	<0.00001^*∗∗*^
ACEI/ARB; *n* (%)	34 (64.15)	10 (18.86)	<0.00001^*∗∗*^
*β*-blocker; *n* (%)	29 (54.71)	12 (22.64)	0.0007^*∗∗*^
Calcium channel blocker; *n* (%)	11 (20.75)	3 (5.66)	0.02^*∗∗*^
Diuretics; *n* (%)	5 (9.43)	7 (13.2)	0.53^*∗∗*^
Nitrates; *n* (%)	11 (20.75)	—	

Data is presented as arithmetic means ± SD (min. ÷ max.); ^*∗*^Student's *t*-test; ^*∗∗*^Chi^2^ test. Explanation of abbreviations used: ACEI: angiotensin-converting enzyme inhibitor, ARB: angiotensin receptor blocker, BMI: body-mass index, PCI: percutaneous coronary intervention, CAD: coronary artery disease, CABG: coronary artery bypass graft, and COPD: chronic obstructive pulmonary disease.

**Table 2:** Lipid profile, concentrations of selected markers of inflammation, oxidative stress, FMD, and IMT in patients and controls before and after treatment with simvastatin.

	CAD group				Controls		
	At baseline	After 6 months	After 12 months	*p*	At baseline	After 12 months	*p*
Number of subjects; females	53 (9)	53 (9)	53 (9)		53 (6)	53 (6)	
Total cholesterol; mmol/L	6.02 ± 0.81^£^	4.54 ± 0.73^¥^	4.97 ± 0.97^§,¶^	0.000001^*∗*^	5.18 ± 0.95	5.26 ± 0.94	0.69^*∗∗*^
LDL cholesterol; mmol/L	3.79 ± 0.76^£^	2.36 ± 0.64^¥^	2.85 ± 0.82^§^	0.000001^*∗*^	2.93 ± 0.73	3.08 ± 0.75	0.46^*∗∗*^
HDL cholesterol; mmol/L	1.29 ± 0.34	1.36 ± 0.46	1.35 ± 0.44	0.32^*∗*^	1.32 ± 0.41	1.27 ± 0.34	0.44^*∗∗*^
Triglycerides; mmol/L	2.11 ± 1.05	1.83 ± 1.1	1.77 ± 0.95	0.06^*∗*^	2.01 ± 1.42	2.2 ± 2.47	0.51^*∗∗*^
Fibrinogen; g/L	3.73 ± 1.02^£^	3.93 ± 1.19	3.85 ± 1.19^¶^	0.65^*∗*^	3.34 ± 0.74	3.26 ± 0.87	0.98^*∗∗*^
hs-CRP; mg/L	2.93 ± 2.53^£^	1.57 ± 1.46^¥^	1.67 ± 1.19^§,¶^	0.021^*∗*^	1.31 ± 1.14	1.34 ± 1.54	0.81^*∗∗*^
TNF-*α*; pg/mL	1.82 ± 1.41^£^	1.66 ± 1.32	1.31 ± 0.95^§,¶^	0.001^*∗*^	0.85 ± 0.45	0.88 ± 0.43	0.67^*∗∗*^
8-Iso-PGF_2*α*_; pg/mg creat.	1305.2 ± 817.1^£^	1333.0 ± 829.5	1285.5 ± 790.3^¶^	0.86^*∗*^	572.6 ± 314.8	547.49 ± 306.8	0.79^*∗∗*^
FMD; %	6.76 ± 2.53^£^	8.25 ± 3.3^¥^	8.12 ± 2.28^§^	0.0004^*∗*^	8.08 ± 2.8	8.26 ± 3.12	0.47^*∗∗*^
IMT; mm	0.7 ± 0.14^£^	0.64 ± 0.14^¥^	0.63 ± 0.16^§^	0.008^*∗*^	0.57 ± 0.08	0.55 ± 0.08	0.27^*∗∗*^

Data is presented as arithmetic means ± SD; Student's *t*-test; Mann-Whitney *U* test; ^*∗*^ANOVA Friedman test; ^*∗∗*^Wilcoxon signed-rank test; ^*¥*^ *p* < 0.05, after 6 months of treatment compared to baseline; ^§^ *p* < 0.05, after 12 months of treatment compared to baseline; ^¶^ *p* < 0.05, after 12 months of treatment compared to controls; ^*£*^ *p* < 0.05, at baseline compared to controls.

**Table 3:** The effect of Q192R and L55M polymorphisms on serum PON1 activity.

	CAD group	Controls	*p*	PON1 activity (U/L)		*p*
	*N* (%)	*N* (%)		CAD group	Controls	
Number of subjects (%)	53 (100)	53 (100)	1.0^*∗∗*^	126.58 ± 85.95	125.94 ± 79.56	0.91^*∗*^

Q192R polymorphism						
Genotype						
QQ	32 (60.4)	31 (58.5)	0.84^*∗∗*^	66.04 ± 9.64	72.28 ± 14.19	0.14^*∗*^
QR	17 (32.1)	18 (34)	0.83^*∗∗*^	196.54 ± 48.24	182.76 ± 40.5	0.43^*∗*^
RR	4 (7.5)	4 (7.5)	1.0^*∗∗*^	302.75 ± 91.65	313.75 ± 72.28	0.85^*∗*^
192R allele carriers	21 (39.6)	22 (41.5)	0.84^*∗∗*^	215.05 ± 70.35	207.71 ± 67.23	0.65^*∗*^

L55M polymorphism						
Genotype						
LL	14 (24.6)	18 (34)	0.39^*∗∗*^	126.13 ± 77.26	136.06 ± 83.35	0.44^*∗*^
LM	31 (58.5)	18 (34)	0.01^*∗∗*^	113.85 ± 68.6	112.8 ± 69.43	0.54^*∗*^
MM	8 (15.1)	17 (32)	0.039^*∗∗*^	163.90 ± 134.64	122.82 ± 95.62	0.74^*∗*^

Data is presented as arithmetic means ± SD; ^*∗∗*^Chi^2^ test; ^*∗*^Mann-Whitney *U* test.

**Table 4:** The effect of Q192R and L55M polymorphisms on serum PON1 activity before and after treatment with simvastatin.

Genotype	CAD group					Controls			
	PON1 activity (U/L)					PON1 activity (U/L)			
	*N*	At baseline	After 6 months	After 12 months	*p*	*N*	At baseline	After 12 months	*p*
All subjects	53	126.58 ± 85.95	125.46 ± 93.13	122.85 ± 87.71	0.65^*∗*^	53	125.94 ± 79.56	128.34 ± 86.8	0.32^*∗∗*^
192QQ	32	66.04 ± 9.64	62.67 ± 9.69	64.6 ± 9.37	0.11^*∗*^	31	72.28 ± 14.19	70.9 ± 16.54	0.23^*∗∗*^
192QR	17	196.54 ± 48.24	204.33 ± 13.74	195.50 ± 18.53	0.7^*∗*^	18	182.76 ± 40.5	193.56 ± 10.12	0.38^*∗∗*^
192RR	4	302.75 ± 91.65	304.25 ± 63.23	287 ± 75.97	0.36^*∗*^	4	313.75 ± 72.28	327 ± 40.52	0.27^*∗∗*^
192R allele carriers	21	215.05 ± 70.35	220.5 ± 14.9	215.83 ± 18.6	0.38^*∗*^	22	207.71 ± 67.23	220.25 ± 16.32	0.22^*∗∗*^
55LL	14	126.13 ± 77.26	126.13 ± 77.26	129.66 ± 25.57	0.76^*∗*^	18	136.06 ± 83.35	151.71 ± 92.07	0.87^*∗∗*^
55LM	31	113.85 ± 68.6	111.87 ± 13.56	104.43 ± 12.4	0.22^*∗*^	18	112.8 ± 69.43	123.58 ± 69.43	0.81^*∗∗*^
55MM	8	163.9 ± 134.64	155.9 ± 40.35	159.00 ± 45.92	0.79^*∗*^	17	122.82 ± 95.62	133.56 ± 105.29	0.45^*∗∗*^

Data is presented as arithmetic means ± SD; ^*∗*^ANOVA Friedman test; ^*∗∗*^Wilcoxon signed-rank test.

**Table 5:** Combined genotypes and PON1 activity.

Genotype	CAD patients					Controls			
	PON1 activity (U/L)					PON1 activity (U/L)			
	*N*	At baseline	After 6 months	After 12 months	*p*	*N*	At baseline	After 12 months	*p*
192QQ/55LL	9	67.77 ± 7.54	66.66 ± 4.79	66 ± 8.21	0.87^*∗*^	8	76.62 ± 10.25	75.75 ± 12.51	0.78^*∗∗*^
192QQ/55LM	20	68.42 ± 9.51	63.47 ± 9.2^¥^	69.42 ± 14.94	0.041^*∗*^	14	76.28 ± 15.17	74.78 ± 17.2	0.44^*∗∗*^
192QQ/55MM	3	55.50 ± 8.69	49 ± 11.12	55 ± 6.24	0.53^*∗*^	10	63.20 ± 12.09	61.60 ± 15.91	0.31^*∗∗*^
192QR/55LL	4	209.80 ± 38.92	221.14 ± 65.69	239 ± 92.44	0.62^*∗*^	6	190.33 ± 41.26	194.2 ± 56.44	0.5^*∗∗*^
192QR/55LM	10	191.72 ± 54.12	197.9 ± 61.48	181.62 ± 56.18	0.88^*∗*^	5	184.6 ± 13.01	200.2 ± 28.73	0.5^*∗∗*^
192QR/55MM	3	192.33 ± 51.73	197.33 ± 48.01	164 ± 60.81	0.13^*∗*^	6	173.66 ± 42.74	187.5 ± 40.25	0.13^*∗∗*^
192RR/55LL	1	233	238	214		2	288 ± 43.84	306.4 ± 43.84	0.18^*∗∗*^
192RR/55LM	1	211	266	230		1	265	254	
192RR/55MM	2	283.5 ± 58.68	356.5 ± 26.16	352 ± 16.97	0.6^*∗*^	1	414	441	

Data is presented as arithmetic means ± SD; ^*∗*^ANOVA Friedman test; ^*∗∗*^the Wilcoxon signed-rank test.

^*¥*^ *p* = 0.05, after 6 months of treatment compared to the baseline value.
